# Green synthesis of *P* − *ZrO*_2_*CeO*_2_*ZnO* nanoparticles using leaf extracts of *Flacourtia indica* and their application for the photocatalytic degradation of a model toxic dye, Congo red

**DOI:** 10.1016/j.heliyon.2022.e10277

**Published:** 2022-08-24

**Authors:** Nichodimus Hokonya, Courtie Mahamadi, Netai Mukaratirwa-Muchanyereyi, Timothy Gutu, Caliphs Zvinowanda

**Affiliations:** a Department of Chemistry, Bindura University of Science Education, P. Bag 1020 Bindura, Zimbabwe; b Department of Physics, University of Zimbabwe, P.O. Box MP 167 Mount Pleasant Harare, Zimbabwe; c Department of Chemical Sciences, Faculty of Science, University of Johannesburg, Doornfontein Campus, P.O. Box 17011, Johannesburg, 2028, Republic of South Africa

**Keywords:** Flacourtia indica, $P-ZrO_{2}CeO_{2}ZnO$ nanoparticles, Reaction mechanisms, Congo red, Photocatalysis

## Abstract

In the present work $P-ZrO_{2}CeO_{2}ZnO$ nanoparticles were synthesised for the first time using phytochemical extracts from *Flacourtia indica* leaves and applied in the photocatalytic degradation of Congo Red in the presence of Light Emitting Diode warm white light. The photocatalytic degradation was optimized with respect to $P-ZrO_{2}CeO_{2}ZnO$ nanoparticle dosage, initial Congo Red concentration, and degradation time. The optimum conditions for $P-ZrO_{2}CeO_{2}ZnO$ nanoparticle synthesis was pH 9, leaves extracts of *F. indica* dosage 4 g 100 mL^−1^, Zirconia, Cerium and Zinc metal ion concentration 0.05 mg/L and metal ion to plant volume ratio of 1:4. The leaves extract dosage, pH and metal concentration had the most significant effects on the synthesis of the nanoparticles. The nanoparticles followed type III physisorption adsorption isotherms with surface area of 0.4593 m^3^g^−1^, pore size of 6.80 nm, pore volume 0.000734 cmg−13 and average nanoparticle size 0.255 nm. A degradation efficiency of 86% was achieved and the optimum degradation conditions were 0.05 g/L of $P-ZrO_{2}CeO_{2}ZnO$ nanoparticle dosage, 10 mg/L initial Congo red concentration, and 250 minutes irradiation time. Data from kinetic studies showed that the degradation followed pseudo first order kinetics at low concentration, with a rate constant of 0.069 min^−1^. The superoxide, $h^{+}$ holes and light were the main determinants of the reaction mechanisms for the degradation of Congo Red. The investigation outcomes demonstrated that $P-ZrO_{2}CeO_{2}ZnO$ nanoparticles offer a high potential for photocatalytic degradation of Congo Red.

## Highlights

• The most significant factors on $P-ZrO_{2}CeO_{2}ZnO$ nanoparticles synthesis were plant leaves dosage, pH and initial metal concentration.
• The nanoparticles exhibited high catalytic activity towards photocatalytic degradation of Congo red.
• Superoxide, $h^{+}$ holes and light were the main determinants of the photocatalytic degradation mechanisms.

## Graphical abstract

**Figure:**
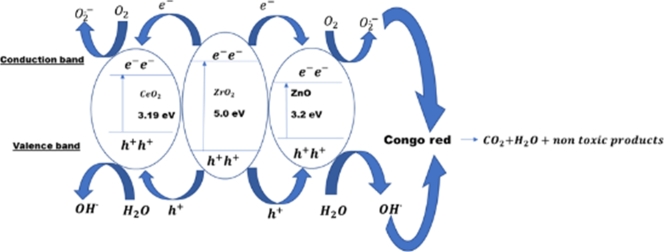

## 1 Introduction

Approximately 1-20% of organic dyes are discharged into the environment after their use in dye-including industries and they pollute huge quantities of water [1]. Dyes find various applications in rubber, textile, plastic, cosmetic, leather, paper making and pharmaceutical industry [2] A large amount of dye material do not bind during the colouration process and is lost to wastewater causing depletion of oxygen and reducing sunlight penetration [3] and this may lead to serious contamination issues and may cause diseases such as cancers [4]. Congo red is a diazo dye which is fairly stable and non-biodegradable due to its complex aromatic structure. It is highly soluble in water and difficult to remove from water. It has carcinogenic properties, hence it has fatal consequences with effects on skin, eyes, reproductive and respiratory systems [5]. Congo red is used in wool, silk, textile and food industries. It is also used in medicine as a biological stain for diagnosis of amyloidosis and an indicator in acidic medium [6].

Congo red has been removed from water using adsorption [7] [8] photocatalytic degradation [9], [10][11][12][13], membrane filtration [14], electrochemical oxidation and bio-treatment [15], sonocatalysis [16], catalytic reduction [17], photo-Fenton process [18], radiation induced degradation [19]. The conventional processes used to treat wastewater contaminated with dyes have proved to be relatively inefficient, expensive and can even cause secondary contamination. Photocatalytic oxidation has attracted many researchers' attention due to its non-toxicity and high efficiency for degrading organic dyes. Hence many efforts have been directed towards synthesis of new catalysts [20]. Metal oxide nanomaterials have attracted much attention as strong candidates for photocatalytic degradation of toxic pollutants [21]. Visible light induced photocatalysis has been shown to be highly efficient in degrading dyes without any secondary contamination [22]. Photocatalytic degradation can be improved by lessening the band gap and extending the absorption range to visible region leading to electron hole separation by coupling semiconductor catalysts [23]. Congo red has been removed from water using photocatalysts such as $TiO_{2}/K$ [24], $LaO_{8}AO.TiO_{3}5\delta (A=Ba,Sr,Ca)$ nanoperovskites [25], $MgZnCr-TiO_{2}$ [26], Ru nanoparticles supported on unfunctionalized single walled carbon nanotubes [27], $Cu_{2}O$ [28], PANI nanoarrays anchored on 2DBiOCl nanoplates [29].

ZnO and TiO2 have been used as effective non-toxic and inexpensive photocatalyst for the effective degradation of a wide range of organic pollutants in recent years. ZnO has an advantage over TiO2 because it absorbs a larger fraction of the UV spectrum [30]. Zinc Oxide is a promising photocatalyst however, its optical absorption is limited in the UV region. However, it has been shown that doping with transition metals alters the photophysical properties and reduces the band gap energy as well as the rate of electron hole pair recombination, resulting in improved catalytic performance [31]. Cerium oxide ($CeO_{2}$) is a semiconducting material with a large exciton binding energy and a bandgap of 3.19 eV. $CeO_{2}$ is used in biosensors, catalysts, electronics, drug delivery and pharmaceutical industry. It is an alternative material for photocatalytic application because of its strong light absorption. $CeO_{2}$ materials have fast electron hole pairs regeneration under light illumination and a longer lifetime [32]. Zirconium dioxide ($ZrO_{2}$) is a ceramic material with a large band gap (c.a 5.0 eV) which allows it to absorb only a negligible fraction of light impinging onto its surface. $ZrO_{2}$ is a low cost material with high redox potentials of photogenerated $e^{-}/h^{+}$ pairs, good optical and chemical stability, biological and environmental compatibility which renders its suitability for photocatalytic reactions. $ZrO_{2}$ has been modified with rare earth metals, transition metals and non-metals in order to achieve charge separation and photocatalytic reactions using lower electronic photons belonging to the visible light spectra [33][34]. Inserting Zirconia into the ceria lattice can improve the lattice oxygen mobility resulting in a better redox quality [35]. Recently doping transition metals with phosphorous atom into the crystal structure has been widely studied. Phosphorous enhances catalytic activity by drawing electrons from metal centres. The d-states at Fermi lever of the electronic structure of metal centre can be modified by the phosphorous atom with abundant valence electrons [36] Nanotechnology has emerged as the most interesting area of research due to the unique properties of nanoparticles which are decided by crucial parameters such as shape, size and morphology [37]. The specific area of nanocomposites is quite interesting due to their enhanced properties such as high surface area to mass ratio which helps in enhancing their absorbing and catalytic ability which in turn aids in removing pollutants from the environment [38][3]. Trimetallic nanoparticles are produced by combining three different metals and catalytic properties can be tailored better than single monometallic catalyst. Their surfaces are unstable and are precipitated away from their solution. However they can be stabilized by block copolymers, organic ligands, surfactants and dendrimers [39]. Trimetallic nanoparticles have higher outstanding catalytic performance when compared with single or bimetallic nanoparticles. Mixing a few metals or alloying also enriches the properties of the metals due to their optimum composition synergic effect between the constituents and structural diversity. The enhanced properties have allowed trimetallic nanoalloys to be applied for various applications. The trimetallic nanoparticles have good charge recombination separation therefore making it a good candidate for photodegradation of organic pollutants [38].

Most physical and chemical methods used to synthesise nanoparticles suffer from several drawbacks such as the use of high pressure and temperature, long reaction time, toxic reagents, requirements of external additives such as specific base, stabilizer and promoter during the reaction which limits the purity of the final product [40]. Physical techniques also require high vacuums, high temperature and relatively expensive equipment. Chemical synthesis drawback is some pollutants and toxic materials are created as products of chemical reactions [41], hence its necessary to use environmentally friendly techniques like green synthesis using plant materials. Plant mediated synthesis of metal nanoparticles using whole or parts of plants is gaining extensive research focus due to its ease in scaling up for larger production, cost effectiveness and environmental friendliness [42]. The bio molecules in plants extract act as electron shuttles in metal reduction whilst others act as capping agents, thereby controlling the aggregation of nanoparticles as well as post surface modification [43]. Few studies have been done on green synthesis of trimetallic nanoparticles and they have been synthesized using *Aegle marmelos* leaves and *Syzgium aromaticum* bud extracts [44], *A membranaceus* [45], leaf extracts of *Eryngium campestre* and *Froriepia subpinnata* [46].

In this study novel P-ZrO_2_CeO_2_ZnO nanoparticles were synthesised for the first time using leaves extract of *F. indica* as reducing agents. F. indica was used to prepare the leaf extract because it has phytochemicals which can readily act as reducing agents for the nanoparticle synthesis and also it was readily available. P-ZrO_2_CeO_2_ZnO nanoparticles were chosen for the photocatalytic degradation of Congo red because it a trimetallic nanoparticle which has the synergic effect brought about by combining the properties of known catalyst such as CeO_2_ and ZnO as well as good charge recombination separation. The mechanism of photocatalytic degradation was determined during this study. The kinetics of the reaction and inhibition reactions were successfully modelled to determine the best conditions for photo-oxidation of the dye. Kinetic modelling included models such as pseudo zero order and second order thus adding a new dimension to photocatalysis kinetic modelling which is commonly studied using the Langmuir-Hinshelwood model only.

## 2 Materials and method

### 2.1 Chemicals and materials

All chemicals used were analytical grade and were used as provided. For synthesizing P-ZrO_2_CeO_2_ZnO nanoparticles the following were used; *Flacourtia indica* leaves, zirconyl chloride octahydrate (Riedel-De Haen Ag Sleeze Hanover), zinc nitrate hexahydrate (Merck, RSA), cerium (IV) sulphate hydrate (Merck, RSA), phosphoric acid (Merck, RSA). For photocatalysis of Congo red using P-ZrO_2_CeO_2_ZnO nanoparticles the following reagents were used; phenol (Riedel-de-Haen. AG), methanol (Avonchem, UK), propanol (ACE, RSA), sodium hydroxide (Glassworld, SA), hydrochloric acid, (Merck, RSA) (Reagent grade), disodium Ethylenediaminetetraacetic acid (Merck, SA), potassium dichromate (Merck RSA), Congo Red (Merck, RSA), and deionised water.

### 2.2 Equipment

The equipment used during the formation of P-ZrO_2_CeO_2_ZnO nanoparticles included; Blender (HE-House) for cutting up the *F. indica* leaves, Muffle furnace (Carbolite, England) for calcining the nanoparticles, Oven (Biobase, China) for drying the nanoparticles. The following equipment were used for characterization of the P-ZrO_2_CeO_2_ZnO nanoparticles; Scanning Electron Microscope (SEM) (Auriga, Zeiss Germany) was used to determine the morphology of the nanoparticles at 200 V and maximum voltage of 40 kV. The $P-ZrO_{2}CeO_{2}ZnO$ nanoparticles were deposited on electron microscopy grids operated at an accelerated voltage of 200 kV with scanning mode and observed. Transmission Electron Microscope (TEM) (Tecnai F20, FEI company, USA) was used to determine the size of nanoparticles suspension on a carbon coated grid and placed in the TEM instrument, and operated at 20-200 kV with a resolution of 2.4 Å. UV-Vis spectrometer (UV Vis) (Thermo-fisher Scientific, USA) was used to follow the optical properties of the nanoparticles during synthesis as well as follow the photocatalysis reaction. Inductively Coupled Plasma Mass Spectrometry (ICP-MS 7800) (Agilent Australia) was used to determine the chemical composition of the nanoparticles. Powder X-ray Diffraction (P-XRD) D2-Phase Diffractometer, (Bruker, Germany) was used to determine the crystallinity of trimetallic nanoparticles with Cu K (∝=1.5406) radiation. The scanning mode used was continuous with a scanning range 2 *θ* from 10 - 90°. Samples were ground into fine powder and placed on sample holder. The surface area was also determined by a Tristar II Plus surface and porosity analyser from Micrometrics (USA). The nanoparticles samples were dried at 80°C for 36 hrs to remove the moisture content prior to analysis. The actual analysis was carried out using an analyser bath temperature of -197.4°C. Orbital shaker (Griffin, German), Attenuated Total Reflectance Fourier Transform–Infra Red Spectrometer (ATR-FTIR, Thermo-fisher scientific) was used to determine the functional groups responsible for reduction of the metal ions at an average of 16 scans, a resolution of 2 cm^−1^ and scanning from 4000-400 cm^−1^. The bare nanoparticles and plant extract were subjected to IR analysis. The equipment used during photocatalytic degradations included Magnetic stirrer (Stuart Scientific) was used to stir the reaction mixture during photocatalysis, pH meter (Adwa AD 1020-Romania) was used for measuring pH during nanoparticle formation. Light Emitting Diode (LED) light Warm white (Panasonic) 20 W, 3000 K, with a power flux of 5.986 x 10^39^ s^−1^ (Power of LED light = 20 watt = work/time = 20 Js^−1^; Energy of photon (E) = hc/*λ* = 6.626 x10^−34^ Js^−1^ x 3.0 x108 ms^−1^/5.95 x10^−9^ m =3.341 x 10^−39^ J; Number of photons emitted per second = 20 Js^- 1^ /3.3408 x 10 ^−39^ J^=^ 5.986 X 1039 s^−1^) was used to provide the light energy during photocatalysis, Centrifuge 5702 R, (Eppendorf) was used to recover the nanoparticles after photocatalysis.

### 2.3 Synthesis of the $P-ZrO_{2}CeO_{2}ZnO$ nanoparticles

The leaves of *F. indica* were collected and washed with deionised water three times to remove dirt and then shade dried for a week. A blender was used to grind the leaves into fine powder. 20 g of the fine plant leaves powder was added to 500 mL deionised water and boiled for 30 minutes on a heating mantle. The mixture was vacuum filtered using a 0.45 μm filter paper to get a clear solution and stored in fridge before use. The obtained extract was used without further dilution [47].

The independent variables that affect the synthesis of the nanoparticles such as pH, plant extract dosage, initial metal concentration and plant extract to metal salt volume ratio [48] were optimized using the Taguchi experimental design. Each metal salt solution (${\mathrm{ZrOCl}}_{2}8H_{2}O$, $\mathrm{Zn}{\left({\mathrm{NO}}_{3}\right)}_{2}6H_{2}0$ and $\mathrm{Ce}{\left({\mathrm{SO}}_{4}\right)}_{2}4H_{2}O$) of the appropriate concentration and pH was added to *F. indica* leaf extract at conditions shown in Table 1 and boiled for 30 minutes; the next salt was then added and subjected to the same conditions. Concentrated hydro phosphorous acid solution (25 mL) was added drop wise to metal salts under vigorous stirring in order to further dope the composite using Phosphorous [36]. The solution was boiled until minimum liquid remains. The semi liquid containing the nanoparticles was oven dried and then the solid was then calcined at 900°C to get the oxide nanoparticles [49]. The optimized conditions were then used to synthesize the nanoparticles before characterisation.

**Table 1:** Taguchi design for the optimization of synthetic conditions of P-*ZrO*_2_*CeO*_2_*ZnO* nanoparticles.

pH	Leaf dosage (g)	metal concentration (M)	Metal to plant extract to
			volume ratio
3	2	0.05	1.2
3	3	0.1	1.4
3	4	0.2	2.1
3	5	0.5	4.1
6	2	0.1	2.1
6	3	0.05	4.1
6	4	0.5	1.2
6	5	0.2	1.4
9	2	0.2	4.1
9	3	0.5	2.1
9	4	0.05	1.4
9	5	0.1	1.2
12	2	0.5	1.4
12	3	0.2	1.2
12	4	0.1	4.1
12	5	0.05	2.1

### 2.4 Photocatalysis procedure

**Figure 1:**
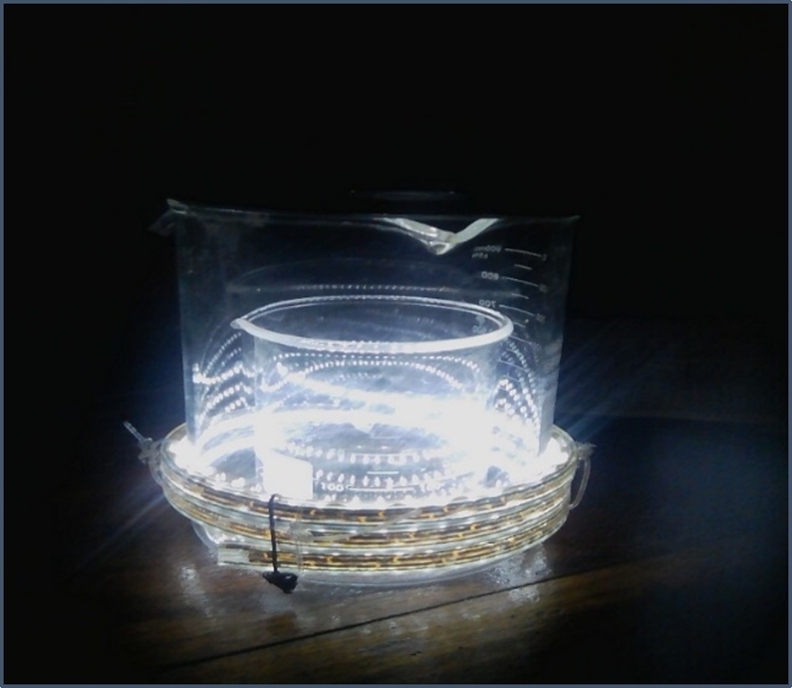
The LED warm white light coiled around the beaker photoreactor.

The photocatalytic activity of $P-ZrO_{2}CeO_{2}ZnO$ catalysts was studied by photodegradation of Congo red as a test dye. The catalyst (1 g/L) was dispersed in 10 mg/L of Congo red and stirred in the dark for half an hour to achieve adsorption-desorption equilibrium before being subjected to UV LED warm white light shown in Fig. 1.

4 mL aliquots were taken at 30 min intervals and centrifuged at 2500 rpm for 5 minutes to remove the suspended catalyst and then analysed by UV-Vis spectrometer at 562.9 nm. The percentage degradation of Congo Red by the catalysts was calculated using the formula in Equation (1),

(1) $$\text{\% degradation}=\frac{C_{o}-C_{t}}{C_{t}}\text{X}\;100$$

$C_{o}$ is the initial Congo red concentration before UV led warm white light illumination, $C_{t}$ is the concentration of Congo red after UV led warm white light illumination at time *t*.

**Figure 2:**
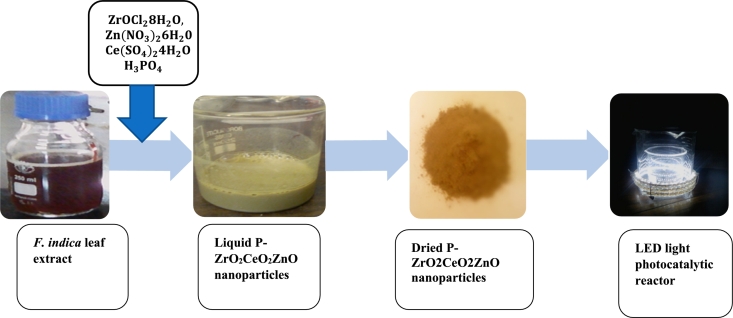
Reaction scheme of the synthesis of *P* − *ZrO*_2_*CeO*_2_*ZnO* nanoparticles up to photocatalysis of Congo red.

Fig. 2 summarizes the green synthesis of the $P-ZrO_{2}CeO_{2}ZnO$ nanoparticles and the photocatalysis of Congo red using the nanoparticles.

## 3 Results and discussion

### 3.1 Optimization of synthetic conditions

The optimization of green synthesis of $P-ZrO_{2}CeO_{2}ZnO$ nanoparticles was carried out using the Taguchi $L_{16}$ orthogonal array design and the results are shown in Table 2.

**Table 2:** Optimization of synthetic conditions of P-*ZrO*_2_*CeO*_2_*ZnO* nanoparticles.

pH	Dosage (g)	metal concentration (M)	volume ratio	Absorbance	Signal/noise ratio
3	2	0.05	1.2	2.00	6.03
3	3	0.1	1.4	1.94	5.76
3	4	0.2	2.1	3.58	11.09
3	5	0.5	4.1	3.68	11.32
6	2	0.1	2.1	0.45	-7.03
6	3	0.05	4.1	1.99	5.98
6	4	0.5	1.2	3.35	10.50
6	5	0.2	1.4	3.97	11.97
9	2	0.2	4.1	4.34	12.75
9	3	0.5	2.1	4.58	13.22
9	4	0.05	1.4	4.83	13.68
9	5	0.1	1.2	3.77	11.52
12	2	0.5	1.4	2.95	9.41
12	3	0.2	1.2	3.66	11.27
12	4	0.1	4.1	3.19	10.07
12	5	0.05	2.1	3.75	11.49

The conditions of the optimum $P-ZrO_{2}CeO_{2}ZnO$ nanoparticles sample obtained using the Taguchi design were pH 9, dosage 4 g/100 mL, metal concentration 0.05 M and volume ratio 1:4. $P-ZrO_{2}CeO_{2}ZnO$ nanoparticles were then synthesized using these optimized conditions and thereafter characterised by various instrumental techniques.

### 3.2 Evaluation of most significant factors and interactions by ANOVA

The data obtained from the synthesis of nanoparticles was analysed by using ANOVA Minitab 18 (see Table 3) to obtain the most important effects and probable interactions between variables. The statistical significance of related effects at 95% confidence level is shown by the probability values less than 5% (p < 0.05). Dosage, pH, and metal ion concentration were significant since the values of p were less than 0.05. However, the *p* value of volume ratio was above 0.05 meaning it was statistically insignificant. The empirical relationship between the tested variables and response (absorbance) was generated using Minitab statistical software is represented by the following Equation (2).

(2) $$\text{Absorbance}=-12.0-1.35P+9.28D-134M+9.7V+0.155P^{2}-0.427D+79.6M^{2}-1.05V^{2}-0.429\mathrm{PD}+3.57\mathrm{PM}+0.222\mathrm{PV}+14.5\mathrm{DM}-2.04\mathrm{DV}+5.\mathrm{MV}$$

where P is pH, D is dosage, M is metal concentration, V is volume ratio.

**Table 3:** ANOVA data from the determination of the most significant factors for nanoparticles synthesis.

Source		DF	Seq SS		Adj SS	Adj MS	F	P
pH		3	8.6173		8.6173	2.87244	25.81	0.012
Dosage (g)		3	4.9617		4.9617	1.65391	14.86	0.026
metal concentration (M)		3	5.6321		5.6321	1.87735	16.87	0.022
volume ratio		3	0.2440		0.2440	0.08135	0.73	0.599
Residual Error		3	0.3338		0.3338	0.11127		
Total		15	19.7890					
S	R-Sq.	R-Sq. (Adj)						
0.3336	98.31%	91.57%						

In equation (2), a positive value represents enhancement relation on response with each term and a negative value implies no enhancement. The synthesis of the nanoparticles is enhanced by dosage or concentration of the leaf extract, volume ratio of the metal to extract concentration, positive interactions between pH, metal concentration, pH and metal concentration, pH and volume ratio dosage and metal concentration as well as metals concentration and volume ratio. In a nutshell the empirical relationship represented by equation (2) shows the effect of various factors on nanoparticle synthesis. From ANOVA results, the value of regression coefficient R-squared is 0.9831 which is close to 1, suggesting the model is appropriate in describing green synthesis of $P-ZrO_{2}CeO_{2}ZnO$ nanoparticles. Dosage or plant extract concentration is significant because at low concentration, unstable nanoparticles are formed and at high concentration segregation can occur.

### 3.3 Main effects of pH, dosage, initial metal concentration and metal to plant extract volume ratio on nanoparticle synthesis

**Figure 3:**
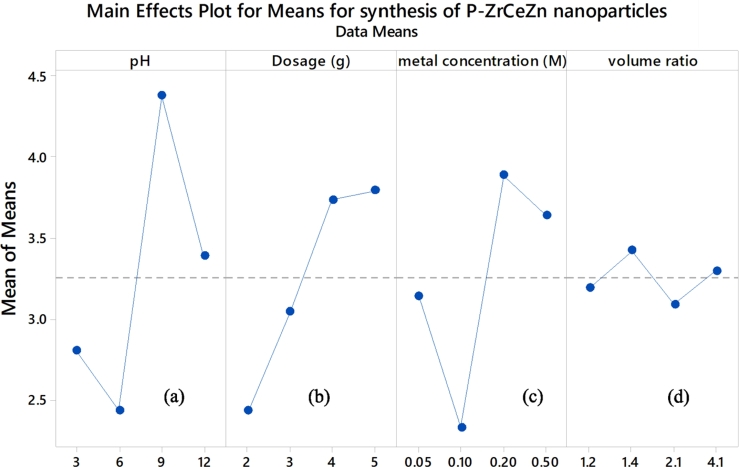
Main effects of (a) pH, (b) dosage, (c) metal concentration and (d) metal to plant extract volume ratio plots for synthesis of P-ZrO_2_CeO_2_ZnO nanoparticles.

The effect of pH, dosage, volume ratio and metal concentration are shown in Fig. 3. The mean response decreases from pH 3 to 6 then starts to rise again at pH 9 and drops at pH 12 assuming all factors are kept constant (Fig. 3a). The influence of pH on the nanoparticle synthesis reaction is shown by its ability to change the electrical charge of the biomolecules in the plant extract which affect their stabilizing and capping abilities, hence the growth of the nanoparticles. Nanoparticles of certain shapes can be preferably formed at a particular pH so that greater stability is achieved. Change in pH can also result in the formation of nanoparticles of different sizes and shapes as well as favouring aggregation of nanoparticles to form larger ones or nucleation to form new nanoparticles [50]. The metal ion reduction process is accelerated by alkaline conditions because under alkaline conditions the *α* glucose (cyclic structure) turns to *β* glucose (open chain structure) which is more reactive since it has an exposed *CHO* group which can readily reduce the metal ions [51].

The mean response was increased as dosage increased assuming all factors are kept constant (Fig. 3b). The concentration of the plant biomass extract used during the nanoparticles synthesis determines the extent of reduction and stabilization which could affect the resulting shapes and sizes of the nanoparticles [52].

For the metal ion concentration, the mean response decreased as metal ion concentration increased from 0.05 to 0.10 M and increases again from 0.1-0.2 M and then start to decrease again from 0.2-0.5 M (Fig. 3c). At low metal ion concentration, the rate of reduction was higher, however, as the metal concentration increased equilibrium was reached and the reducing agent became the limiting factor. The formation of nanoparticles only happened when the plant extract and metal were within suitable range for nucleation (Fig. 3d). The rate of nucleation was mainly affected by the availability of the capping and reducing agents as they influenced whether the metal precursors were to be reduced [53]. Hence, low metal precursor and high plant extract favoured nanoparticles formation probably due to an increased electron density as charged groups in the reductants increased.

### 3.4 Possible reaction mechanism for the synthesis of $P-ZrO_{2}CeO_{2}ZnO$ nanoparticles

*F. indica* leaves have alkaloids, flavonoids, aldehydes, ketones, carboxylic acid ester and phenolic compounds which are all potential reducing and stabilizing agents for nanoparticles synthesis due to their free *π* electrons or hydroxyl groups. Metal salts containing Zinc, zirconia and cerium are reduced to $Zr^{0}$, $Ce^{0}$ and $Zn^{0}$ nanoparticles by the reducing agents and free electrons from the leaves extract of *F. indica* [54]. The first possible reaction is complexation between the phytochemicals in the *F. indica* leaves and the metal salts, then the hydroxyl groups of the phenolic compounds and flavonoids would combine with the zinc, zirconia and cerium ions to form a transitional complex. Electrons will be transferred to the metal ion to form the zero valent nanoparticle Equations (3), (4) and (5).

(3) $$Zr^{4+}+ROH+4e^{-}\;\to \;Zr^{0}$$

(4) $$Ce^{2+}+ROH+2e^{-}\;\to \;Ce^{0}$$

(5) $$Zn^{2+}+ROH+2e^{-}\;\to \;Zn^{0}$$

The zero-valent phytochemical stabilized nanoparticles were then calcined at 900°C to from the oxide nanoparticles in Equations (6), (7) and (8).

(6) $$Zr^{0}\;\to \;ZrO_{2}$$

(7) $$Ce^{0}\;\to \;CeO_{2}$$

(8) $$Zn^{0}\;\to \;ZnO$$

Reducing sugars can reduce metal ions into zero valent nanoparticles directly and other sugars are first hydrolysed into their component reducing sugars. They provide the electrons to reduce the $Zr^{4+}\;Ce^{2+}$ or $Zn^{2+}\;{}^{+}$ to $Zr^{0}$,$Ce^{0}$ and $Zn^{0}$ under basic conditions respectively. Nucleophilic addition of $OH^{-}$ group to the aldehyde group results in oxidation to the carboxyl group which then reduces the metal ions to nanoparticles

### 3.5 Characterisation of nanoparticles

**Figure 4:**
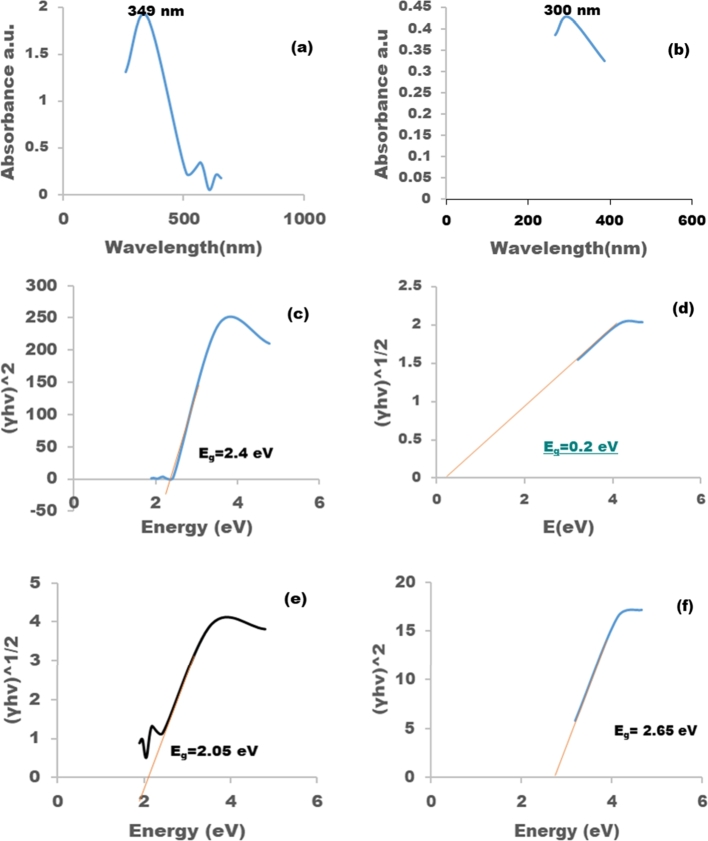
UV Vis spectrum of a) *P* − *ZrO*_2_*CeO*_2_*ZnO* nanoparticles and b)*ZrO*_2_*CeO*_2_*ZnO* nanoparticles and determination of band gap c) and d) for *ZrO*_2_*CeO*_2_*ZnO* nanoparticles and e) and f) for *P* − *ZrO*_2_*CeO*_2_*ZnO* nanoparticles.

The *F. indica* mediated synthesis of $P-ZrO_{2}CeO_{2}ZnO$ nanoparticles were monitored through colour changes from brown to yellowish after half an hour of reaction time. The intrinsic property of nanoparticles is surface plasmon resonance and it results in colour changes. The optical properties of the synthesized $P-ZrO_{2}CeO_{2}ZnO$ was monitored using UV Vis spectroscopy in the range 300 to 900 nm is shown in Fig. 4. The UV Vis spectra of the $P-ZrO_{2}CeO_{2}ZnO$ nanoparticles (Fig. 4a) peaked at 349 nm and or the $ZrO_{2}CeO_{2}ZnO$ nanoparticles (Fig. 4b) peaked at 300 nm. The UV diffuse reflectance spectroscopy (UV-DRS) was carried out to determine the bandgap of the both nanoparticles. The optical band gap was determined using the Tauc's law equation of the absorption coefficient (∝) and the photon energy E (hv) expressed as:

$$\propto =\text{A}{\left(E_{g}-\mathrm{hv}\right)}^{n}/\mathrm{hv}$$

Where A is a constant, n=1/2 is a directly allowed transition, n=2 is an indirectly allowed transition [47]. The optical band gap energy was determined by plotting (∝hv)^n^ vs hv as shown in Fig. 4c-f. The experimentally determined bandgap of the$ZrO_{2}CeO_{2}ZnO$ nanoparticles was 2.4 eV as shown in Fig. 4c and for the$P-ZrO_{2}CeO_{2}ZnO$ nanoparticles the bandgap was 2.65 eV as shown in Fig. 4f. The results show that phosphorous doping was able to reduce the band gap which is beneficial for photocatalysis.

**Figure 5:**
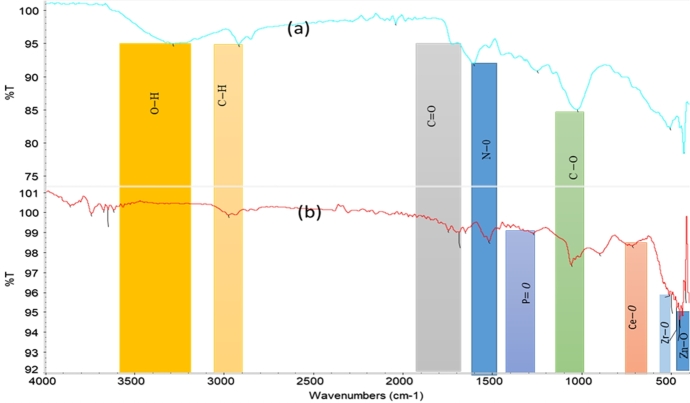
FT-IR Spectrum of (a) leaves of *F. indica* (b) *P* -*ZrO*_2_*CeO*_2_*ZnO* nanoparticles.

The effect of phytochemicals in green synthesis of $P-ZrO_{2}CeO_{2}ZnO$ nanoparticles was determined by FTIR analysis. The FTIR spectrum of the raw plant leaves is shown in Fig. 5a. The presence of the −OH group in the leaf extract is attributed to phenolic compounds and is confirmed by a band at 3285 cm^−1^ [55] and a medium band at 2917 cm^−1^ represent the C−H stretch of alkanes [56]. A small band at 1706 cm^−1^ is due to the C=O stretch of *α* and *β* unsaturated aldehydes and ketones [57] and another C=O at 1743 cm^−1^ [58] amide 1 band at 1649 cm^−1^ [59], and the other small band at 1024 cm^−1^ is due to the C−O of carboxylic acid ester [60]. The $P-ZrO_{2}CeO_{2}ZnO$ nanoparticles (Fig. 5b) have extra bonds which appear in the region below 1000 cm^−1^ and these can be attributed to formation of metal oxide nanoparticles, Ce−O appears at 712 cm^−1^ [61] and at 508 cm^−1^ [62], Zr−O stretching band at 508 cm^−1^ [63] and Zn−O at 493 cm^−1^ [64] [65] and another Zn−O at 712 cm^−1^ [61]. The location of bands in $P-ZrO_{2}CeO_{2}ZnO$ nanoparticles is different from those of the leaves extract of *F. indica* demonstrating a proper linkage between functional groups present in leaves extract of *F. indica* and nanoparticles [66]. The FT-IR spectrum of the raw leaves shows phenolic O−H band at 3285 cm^−1^ which is suppressed in $P-ZrO_{2}CeO_{2}ZnO$ nanoparticles and appears at 3616 cm^−1^ due to free hydroxyl such as adsorbed water [67], the flavonoids, reducing sugars aldehydes and ketones C=O band shift is at 1706 to 1682 cm^−1^ due to unsaturated aldehydes and ketones [68], N−O band shift, 1514 to 1515 cm^−1^, C−O band shift, 1024 to 1055 cm^−1^ [42] or v(Ce−O−Ce) vibration [40]. The band at 1267 cm^−1^ can be attributed to the P=O phosphoryl bond due to doping [69] [70]. The band shift in C−O and the absence of the intermolecular bonded O−H band indicates that these groups participated in nanoparticle synthesis. The FT-IR results indicate that the phytochemicals in *F. indica* can reduce the metals and stabilize them during synthesis.

**Figure 6:**
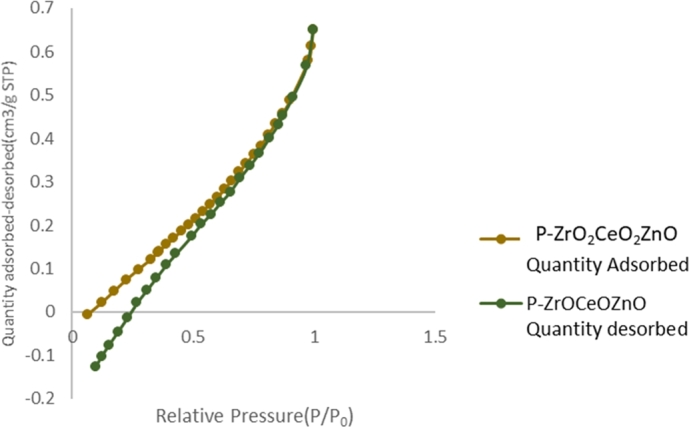
N_2_ adsorption-desorption curves of P-*ZrO*_2_*CeO*_2_*ZnO* nanoparticles.

BET studies were carried out to study the surface area, pore volume and diameter of the nanoparticles. The physisorption isotherms for the P-ZrO_2_CeO_2_ZnO nanoparticles shown in Fig. 6 exhibited Type III isotherm which is obtained when interactions between the adsorbent and adsorbate are weak, the material has no identifiable monolayer formation and the adsorbed molecules are clustered around the most favourable sites on the surface of the nonporous solid [71].

The $P-ZrO_{2}CeO_{2}ZnO$ nanoparticles have a surface area of 0.4593 m^2^g^−1^, pore size is 6.80 nm, pore volume of 0.000734 cm^3^g^−1^ respectively. The pore sizes are in the range of mesoporous materials (2-50 nm diameter) making the $P-ZrO_{2}CeO_{2}ZnO$ catalyst suitable for adsorption and interaction of the Congo Red on the catalyst surface [84].

**Figure 7:**
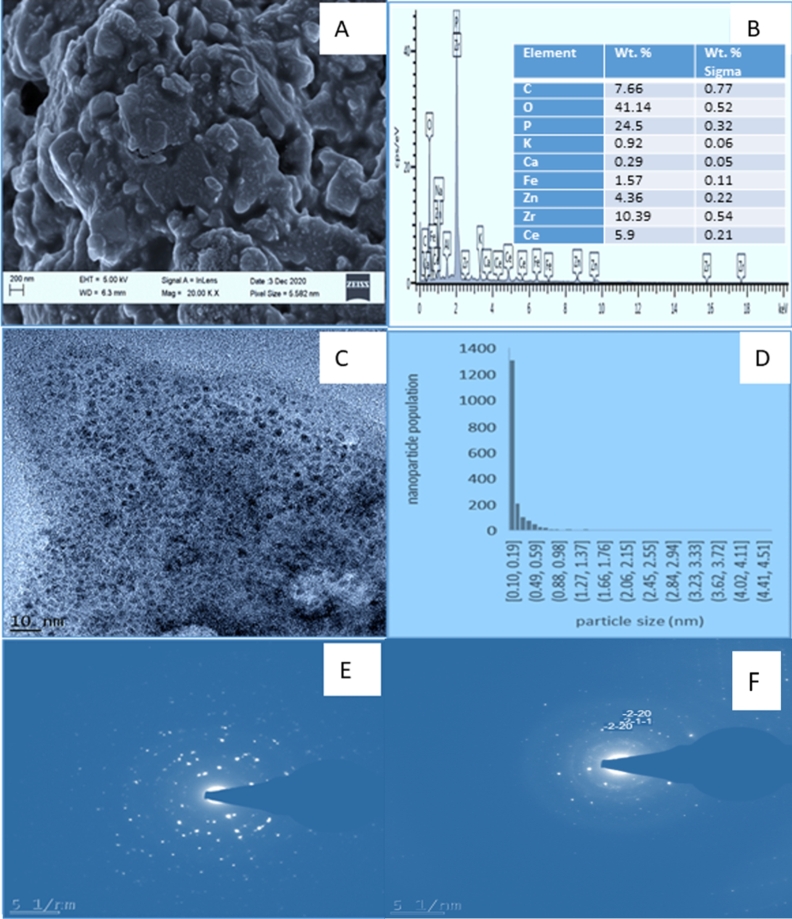
(a) SEM image, (b) EDS spectrum, (c) TEM image, (d) Particle distribution (e) SAED image of P-*ZrO*_2_*CeO*_2_*ZnO* (f) Indexed SAED image of P-*ZrO*_2_*CeO*_2_*ZnO*.

**Figure 8:**
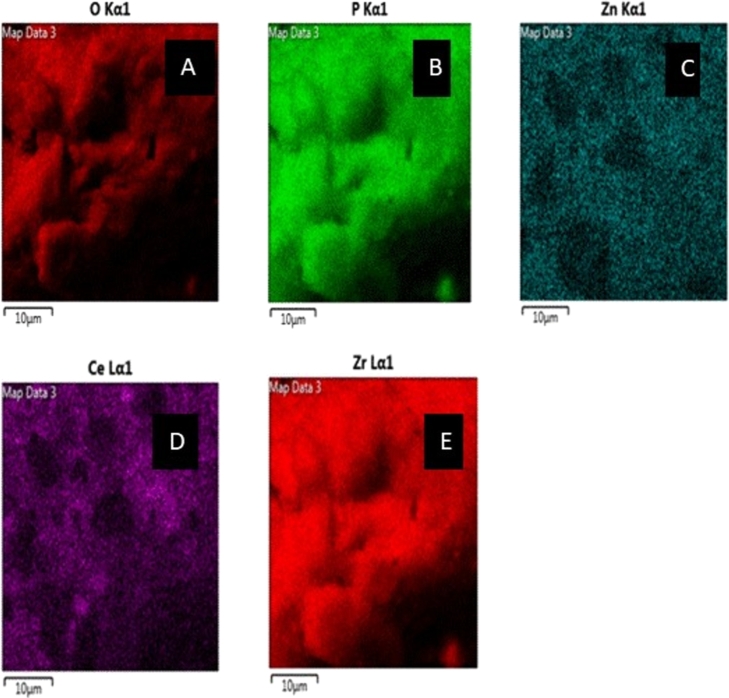
Electron mapping images a) Oxygen, b) Phosphorous, c) Zinc, d) Cerium, e) Zirconium within the P-*ZrO*_2_*CeO*_2_*ZnO* nanoparticles.

The synthesized nanoparticles morphology and surface nature was determined by SEM as shown in Fig. 7a. The SEM images show that the nanoparticles have small irregular shaped particles embedded within flake like structures. SEM-EDX was used to determine the elemental composition of the synthesised nanoparticles and the results showing the percentage weight are shown in Fig. 7b. The EDX elemental mapping confirmed the presence of Carbon (7.66%), oxygen (41.14%), phosphorous (24.5%), potassium (0.92%), Calcium (0.29%), iron (1.57%), zinc (4.34%), zirconium (10.39%) and cerium (5.9%). The peaks for carbon, oxygen, potassium, calcium, iron were from impurities present in the leaves extract of *F. indica* and metal salts used to synthesize the nanoparticles. The presence of oxygen confirms that oxides were formed during nanoparticle synthesis. Transmission Electron Microscopy (TEM) and Image J software were used to determine the particle size as shown by TEM images in Fig. 7c and particle size distribution plots in Fig. 7d. The $P-ZrO_{2}CeO_{2}ZnO$ nanoparticles demonstrate some dispersion and a few clusters with particle size ranging from 0.10 - 4.51 nm. Most of the nanoparticles were within the 0.10 - 0.59 nm diameter range. The average nanoparticle size was 0.255 nm. The particles are small with appearance of some large particles probably due to some plant tissues which were calcined together with the nanoparticles. The TEM images further confirmed that the nanoparticles had irregular shape, hence, TEM results correlated with SEM results in terms of the shape of the nanoparticles. The Selected area electron diffraction (SAED) pattern was also recorded using HR-TEM as shown in Fig. 7e. The ring like structure of the SAED pattern confirms the polycrystalline nature of the nanoparticles [72]. The SAED image was also indexed using Odpin online software diffraction pattern indexing at a(Å) = 4.05, b(AA) = 4.05 and c(Å)= 4.05 as shown in Fig. 7f and ∝, *β* and *γ* = 90^∘^ with a diffraction constant of 440. The first circle had (hkl)_1_ corresponding to 211 which can be indexed to 2*θ* =60.10 of ZrO_2_. The results from SAED indexing suggest that zirconia and cerium oxide nanoparticles were formed. The two other circles had (hkl)_1_ corresponding to 220 which can be indexed to 2*θ* =54.54 of CeO_2_. The selected area electron mapping of the nanoparticles is shown in Fig. 8a oxygen, 8b phosphorous, 8c zinc, 8d Cerium and 8e zirconium. The electron images were evenly distributed except for some minor spaces indicating the inclusion of the elements in nanoparticle synthesis. The elemental composition of the raw leaf extract and the nanoparticles was determined by ICP-MS. There was a general increase in the amount the heavy metals in P - ZrO_2_CeO_2_ZnO nanoparticles as compared to the *F. indica* leaf extract and the increases were 173.2 to 6363.2 mg/g for Zn, 5.89 to 3458.48 mg/g for Zr and 12.75 to 2957.64 mg/g for Ce. These results support the fact that the target metals were successfully incorporated in the $P-ZrO_{2}CeO_{2}ZnO$ nanoparticles composite.

**Figure 9:**
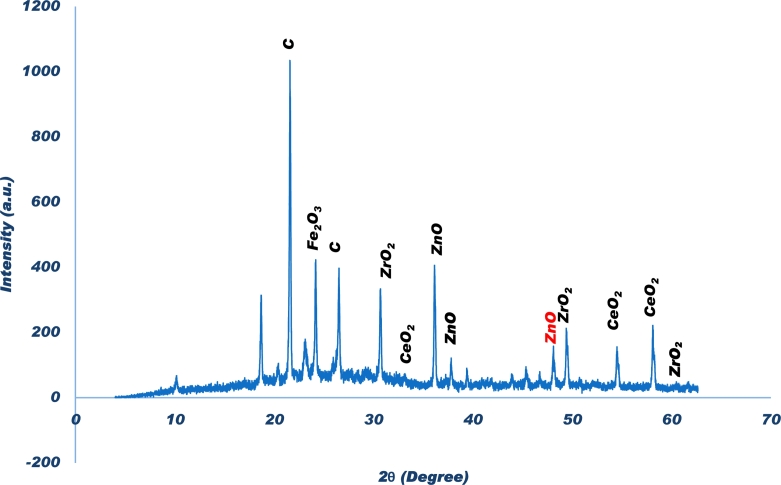
XRD pattern of the P-*ZrO*_2_*CeO*_2_*ZnO* nanoparticles.

The crystalline nature, phase identification and composition analysis of the $P-ZrO_{2}CeO_{2}ZnO$ nanoparticles was determined using Powder X-ray Diffraction (P-XRD) and Profex software version 4.2.4. The P-XRD patterns are shown in Fig. 9 and they can be indexed to the pure wurtzite structure of bulk *ZnO* (Joint Committee on Powder Diffraction Standards, JCPDS NO. 36-1451). The characteristic diffraction peaks were observed at 2*θ* = 36.33, 37.84, 48.17 were indexed to the Bragg reflections (002),(101),(102) respectively, which are planes of the wurtzite structure of *ZnO* [73], [89] [74], [92]. The diffraction peaks at 2*θ* =30.19, 50.25, 60.10 were indexed to the Bragg reflections (101), (112), (211) respectively and can be attributed to the monoclinic phase of $ZrO_{2}$ (JCPDS No. 01-0731523) [93][75][95]. $CeO_{2}$ was also present in the nanoparticle composite and its presence was shown by diffraction peaks at 2*θ* = 28.5, 33.08, 54.54, 59.08, indexed to the (111), (200), (220), (222) face centred cubic structure of $CeO_{2}$ (JCPDS No. 34-0394) [76]. The peak at 2*θ* = 21.59 and 26.51 can be indexed to the amorphous Carbon in the nanoparticles[77] [78]. The peak at 2*θ* =24.16 can be indexed to the (012) of Fe_2_O_3_ [79].

The crystalline nature of the $P-ZrO_{2}CeO_{2}ZnO$ nanoparticles was confirmed by both P-XRD and SAED analysis. XRD analysis also showed the presence of oxides after calcination of the $P-ZrO_{2}CeO_{2}ZnO$ nanoparticles.

The crystalline size was calculated using the Debye - Scherrer's formula (Equation (9)), [80],

(9) $$d=\frac{0.94\lambda }{\beta cos\theta }$$

where *λ* is the X ray wavelength (1.5406 Å), *β* is the full half width maximum of the most intense peak and *θ* is the Bragg's angle position. 2*θ* = 36.2 was used to calculate the crystalline size of ZnO nanoparticles and the size was 0.247 nm. 2*θ* = 30.198 was used to calculate the crystalline size of $ZrO_{2}$ and the size was 0.296 nm. 2*θ* = 47.48 was used to calculate the crystalline size of $CeO_{2}$ and the size was 0.191 nm. The crystalline sizes of the nanoparticles determined by P-XRD calculated using the Debye-Scherrer's formula support the TEM results since nanoparticle size range in the same interval. ICP-MS, EDX and P-XRD data suggest that phosphorous, zirconia, cerium and zinc were successfully incorporated during nanoparticle synthesis. All the techniques from UV Vis, FT-IR, SEM, TEM P-XRD, ICP-MS, SAED and elemental imaging maps help to suggest that nanoparticles were produced during the process.

The optimization of the synthesis of $P-ZrO_{2}CeO_{2}ZnO$ was compared with other studies and the results are shown in Table 4. The nanoparticles compared well with others in terms of size and time taken to synthesize the nanoparticles.

**Table 4:** Comparison of synthesis of P-ZrO_2_CeO_2_ZnO nanoparticles with other studies.

Reducing agent	Nanoparticles	Size/nm	Morphology	Possible molecules responsible for synthesis	Synthetic conditions	References
Erngium campestre/ Froriepia subpinnata	Cu/Cr/Ni	10.96-18.73	cubic, plate	flavonoids, phenolic acid	time 3 min, temperature 20-70°C, extract volume ratio (0.5-3.0)	[46]
trisodium citrate/ microwave	Au/Pt/Ag	20-51.3	nanowire and aspheric	citrate	microwave at 320 W, cycle 15 s ON, OFF 5 s for 6 min	[81]
Aegle marmelos leaf	AgAuPd	8-11			10 min, ambient temperature	[44]
microwave/trisodium citrate	La/Cu/Zr carbon quantum dots	30-100	fibrous	citrate	100 W for 5 min for carbon, 400 W for 2 min (10 s ON 5 s) oven dried for 1 hr.	[38]
*Flacourtia Indica* leaves	*P* − *ZrO*_2_*CeO*_2_*ZnO*	0.10-4.51	cubic	Flavonoids, tepernoides, aldehydes, ketones, reducing sugars.	pH 9, dosage 4 g/100 ml, metal concentration 0.05 M, metal to plant volume ratio 1:4, boil for 30 minutes.	This study
*1.2 hexadecanediol*	PtNiFe/C	5.3-7.5	-	Octylethersolvent, oleylamine, oleic acid as capping agent	Thermally heat at 280°C under O_2_ then at 400-800°C under H_2_	[82]
*Oleylamine/oleic acid*	NiPdPt/C	30-52	Truncated octrahedra	-	Heated under nitrogen, mixed with Vulcan carbon in hexane and sonicated	[83]
*Abscorbic acid*	Ag/Au/Pd	32-33			Adscobic acid added whilst stirring	[84]
*Castor oil polyol*	Sr_0.3_Mg_0.7_Fe_2_O_4_	68	nanocubic		Reflux with continuous stirring for hr. at 120°C followed by coolin for 1hr at 27°C	[85]
*Platycodon grandiflorum* root extract	FeAgPt	10-20	spherical	Carbohydrates,	Sonicated after adding of each salt to root extract	[86]
				proteins, lipids, saponins, triterpenoids		

### 3.6 Catalytic activity of $P-ZrO_{2}CeO_{2}ZnO$ nanoparticles on degradation of Congo red

The catalytic activity of $P-ZrO_{2}CeO_{2}ZnO$ nanoparticles was evaluated for the degradation of Congo red under UV led light. The parameters such as quantity of catalyst, initial concentration of Congo Red and degradation time was optimized.

#### 3.6.1 Effect of catalyst amount

**Figure 10:**
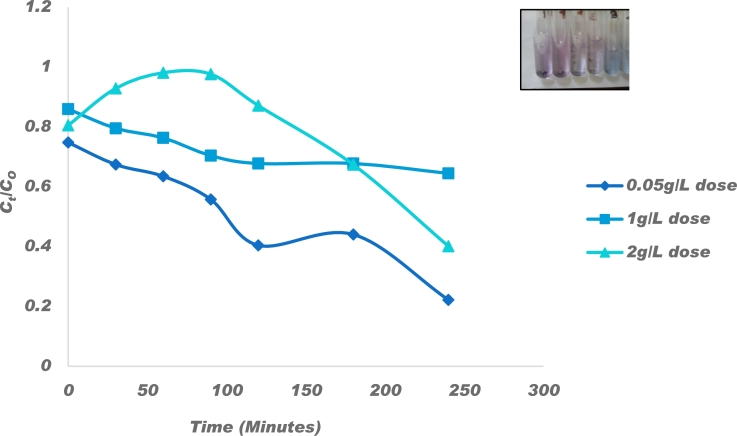
Effect of 0.05, 1 and 2 g/L catalyst dosage on 10 mg/L Congo red decolouration and insert colour changes with time after decolouration.

The effect of the amount of catalyst was determined by varying the dosage of $P-ZrO_{2}CeO_{2}ZnO$ nanoparticle catalyst (0.5, 1.0 and 2.0 g/L), keeping the concentration of Congo Red fixed at 10 mg/L and the results are shown in Fig. 10. Quantitative determination of Congo Red remaining in solution was determined by UV-vis spectrophotometry. The absorption efficiencies before light was illuminated were 32.6%, 20.46% and 7.13% and these increased to 77.82%, 59.90% and 35.55% for catalyst loading 0.5, 1.0 and 2.0 g/L, respectively, after illumination. Aboutaleb and El-Salamony [87], observed a similar trend whereby an increase in catalyst loading resulted in a decrease in removal efficiencies. The decrease in decolouration efficiencies can be ascribed to reduction in active sites as they became saturated. The increase in decolouration at low dosage was due to the amount of catalyst surface available for absorption of the Congo Red molecules and higher absorption of photon energy which leads to further formation of reactive free radicals and dye decomposition. Higher catalyst concentration results in lower rate of decolouration due to the accumulation of particles which leads to dispersion and reduced penetration of light and subsequently limited decolourization [37].

#### 3.6.2 Effect of initial Congo red concentration

**Figure 11:**
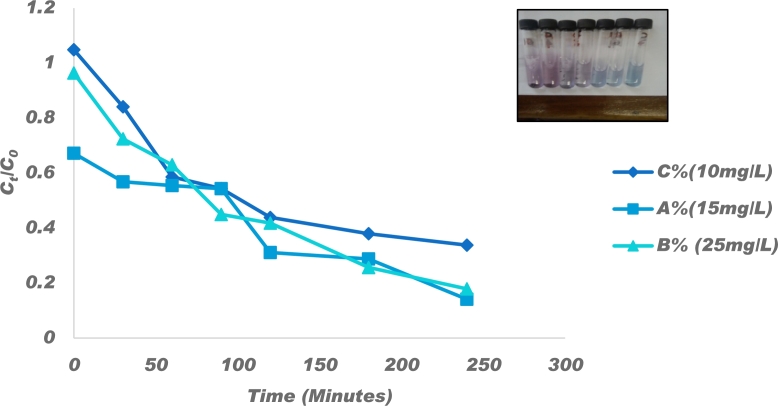
Effect of 10, 15 and 25 mg/L Congo red changing concentrations on decolouration using 1 g/L of*P* − *ZrO*_2_*CeO*_2_*ZnO* nanoparticles and insert colour changes as the reaction progressed.

The effect of Congo Red dye concentration on its photocatalytic degradation was studied by varying the dye concentration from 10, 15 and 25 mg/L of Congo Red at 1 g/L catalyst loading and the results are as shown in Fig. 11. The absorption efficiencies were 27.57%, 43.17% and 15.87% before light was illuminated and the removal efficiencies were 85.85%, 82.07% and 66.19% for concentrations 10 mg/L, 15 mg/L and 25 mg/L, respectively. A similar trend was observed by Shekardasht et al. [88] who suggested that more light exposure would be required for higher concentrations of Congo red. When the dye concentrations were low, the removal efficiencies were very high due to high absorption-desorption equilibria. When the concentration of the dye increased, more Congo red molecules, intermediates and photoproducts competed for absorption onto the active sites of the catalyst surface leading to an effective reduction in the reaction rate [89].

#### 3.6.3 Effect of degradation time

**Figure 12:**
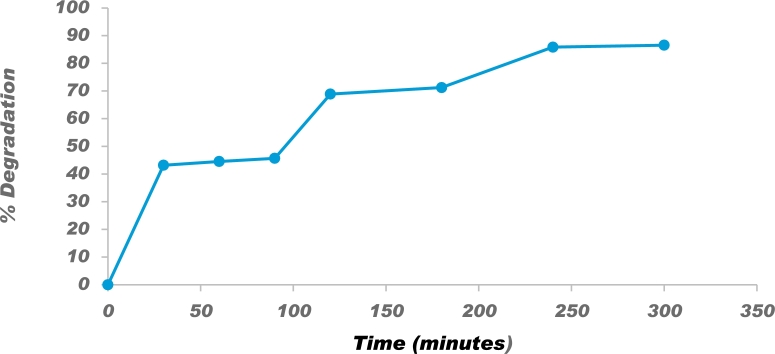
Effect of contact time on photocatalytic degradation of 15 mg/L Congo red using 1 g/L P-ZrO_2_CeO_2_ZnO nanoparticles.

The reaction time was optimized using 1 g/L of $P-ZrO_{2}CeO_{2}ZnO$ nanoparticles, 100 mL of 15 mg/L of Congo red and the reaction was monitored every 30 min in the range 30-300 min. The experimental results are shown in Fig. 12 and these indicate an optimum time of 250 min for the catalytic degradation of the dye. Arunadevi et al. [90] reported at optimum time of 180 min using Cd,Ba−CuO nanoparticles. In similar studies, an optimum degradation time of 180 min was reached using $Fe_{2}O_{3}-CeO_{2}$ nanoparticles as photocatalyst [87]. The longer degradation time experienced in this experiment could be due to low strength of the light source and clamping of the photocatalyst.

### 3.7 Reaction kinetics

Kinetic study is crucial because it describes the rate of Congo red degradation which gives an indication of the amount of time required for the reaction to be completed. The decolourization of Congo red was evaluated using the pseudo zero order, Langmuir-Hinshelwood (L-H) and pseudo second order kinetic models. The pseudo zero order kinetic model assumes that the change in concentration with time is effectively linear and is represented by Equations (10), (11) & (12).

(10) $$\text{Rate}=-\frac{d[A]}{dt}=k_{f}{\left[A\right]}_{0}{\left[R\right]}_{0}=k_{app}$$

(11) $${\left[A\right]}_{t}={\left[A\right]}_{0}-k_{app}t$$

(12) $$k_{app}=k_{f}{\left[A\right]}_{0}{\left[R\right]}_{0}$$

The Langmuir-Hinshelwood (L-H) model assumes that at trivial initial concentration the catalytic reaction follows pseudo first order kinetics [91]. The pseudo first order kinetic model assumes that the number of degrading molecules is small relative to the catalyst population, therefore the rate of change of concentration with time is directly proportional to the concentration of Congo Red remaining in the system. The pseudo first order kinetics rate equation (Equation (13)) [92] was used to analyse the removal rate,

(13) $$r=-\frac{dc}{dt}=k_{app}t$$

and the integral form is shown in Equation (14).

(14) $$\frac{lnC_{o}}{C_{t}}=-k_{app}$$

where $C_{0}$ is the initial concentration, *C* is the concentration at time *t*, $k_{\mathrm{app}}$ is the apparent 1^st^ order rate constant which is determined from the slope of the plot of ln $\frac{C_{0}}{C}$ vs *t* and it also gives the rate of photocatalytic degradation (min^−1^) and the higher the rate the faster the reaction [93]. The half-life for the pseudo first order reaction is calculated using Equation (15).

(15) $$t_{\frac{1}{2}}=\frac{ln2}{k}$$

For a pseudo second order kinetic model the rate of concentration change is proportional to the square of the concentration at that particular instant [94], the differential equation for a second order photokinetic degradation is shown in Equation (16).

(16) $$\frac{dC(t)}{dt}=-k_{2}C^{2}(t)$$

The pseudo second order model is represented by Equation (17)

(17) $$\frac{1}{C_{0}}\;-\frac{1}{C(t)}=k_{2}t$$

where $C_{0},C_{t}$ and $k_{2}$ are the initial concentration, equilibrium concentration and second order apparent rate constant respectively. A plot of *t*/$C_{t}$ vs *t* gives a straight line, and the gradient of the slope is used to determine the rate constant.

**Figure 13:**
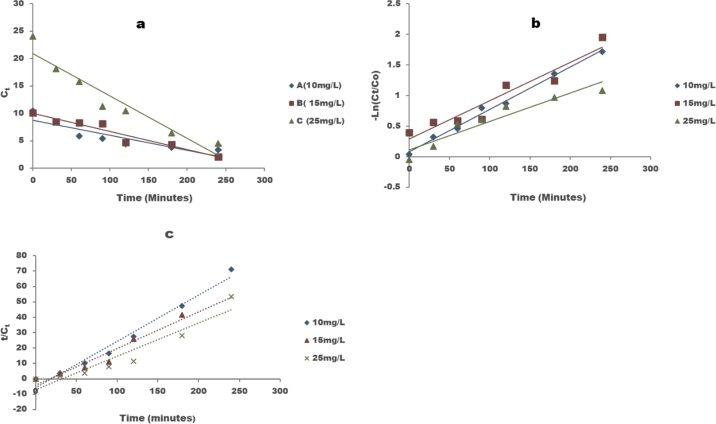
a) Pseudo zero order, b) Pseudo first order, c) pseudo second order degradation kinetic model for 10, 15 and 25 mg/L Congo red using 1 g/L P-ZrO_2_CeO_2_ZnO nanoparticles.

The results of reaction kinetics study were modelled using pseudo zero, pseudo first and pseudo second order kinetic models as shown in Fig. 13 a, b and c respectively and the apparent rate constant and reaction rates are shown in Table 5. At low concentration (10 mg/L) the reaction best fitted pseudo first order kinetics with rate constant 0.0069 min^−1^ and a similar trend was observed by Boudiaf et al. [95], Zhang and Yan [28] and Vattikuti et al. [96]. Pseudo first order reactions are heterogeneous photocatalysis and adsorption-desorption process on the photocatalyst is not disturbed by decomposing reactions [97]. Pseudo first order kinetics means the rate of reaction depends only on the isolated reactant in this case Congo Red since a difference in concentration of the reactant in excess will not affect the reaction. At 15 mg/L the reaction followed pseudo second order kinetics with R^2^ = 0.9401 with a rate constant of 0.2376 min^−1^ and at higher concentration of the dye (25 mg/L), the reactions followed zero order kinetics and the rates constant was 0.0775 min^−1^. Zero order kinetics implies that the reaction rate was constant and independent of the concentration of Congo red and other reacting species.

**Table 5:** Parameters of kinetic study of the photocatalytic degradation of Congo red.

	Pseudo zero order		Pseudo first order			Pseudo second order	
Initial concentration (mg/L)	*K*_*app*_ (min^−1^)	*R*^2^	*K*_*app*_ (min^−1^)	*R*^2^	*t*_1/2_	*K*_*app*_ (min^−1^)	*R*^2^
10	0.0275	0.7999	0.0069	0.992	100.46	0.3016	0.9773
15	0.033	0.9296	0.0063	0.915	110.02	0.2376	0.9401
25	0.0775	0.9141	0.0046	0.899	150.68	0.217	0.9003

### 3.8 Regeneration of the catalyst

**Figure 14:**
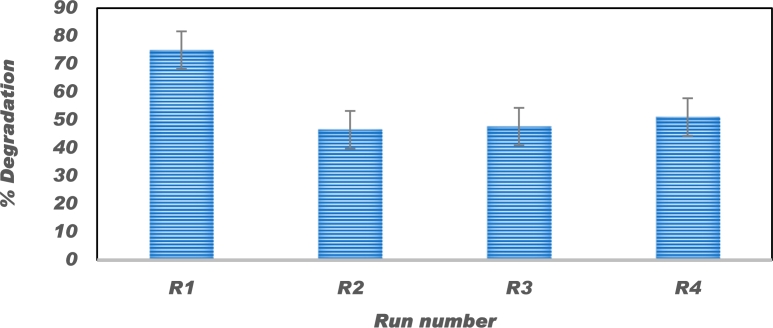
Four recycling experiments for decolourisation of 20 mg/L Congo red using 1 g/L *P* − *ZrO*_2_*CeO*_2_*ZnO* nanoparticles.

The regeneration of the catalyst was investigated by running experiments at 20 mg/L Congo red concentration, catalyst dose 1 g/L, and uncontrolled pH for 4 cycles using the same catalyst. The used catalyst was recovered by centrifugation and washed with distilled water followed by drying at 110°C. The extraction efficiencies obtained were 75%, 46.6%, 47.7% and 51.1% for the cycle 1, 2, 3, 4, respectively as shown in Fig. 14. The general decrease in photocatalytic activity of the $P-ZrO_{2}CeO_{2}ZnO$ catalyst can be ascribed to photo-corrosion under light irradiation and loss of some weakly bound nanoparticles on the catalyst surface [98]. The slight increase between the third and fourth cycle can be due to the fact that during catalyst recovery trace amount of the dye can remain on the catalyst surface hence it has a cumulative effect on the overall concentration of the dye when fresh dye is added during each cycle.

### 3.9 The catalytic reaction mechanism

**Figure 15:**
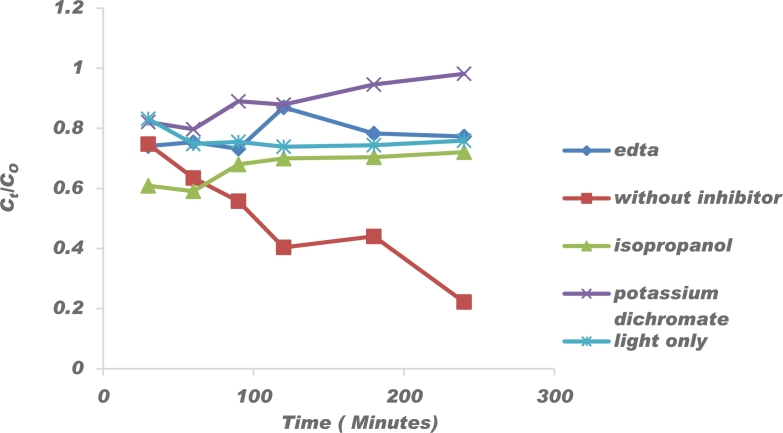
Effect of different scavengers on degradation of 15 mg/L Congo red.

The catalytic reaction mechanism of the Congo red degradation was determined by scavenging experiments with a) no scavenger, b) 1 mM Ethylenediaminetetraacetic acid c) 1 mM potassium dichromate d) 1 mM isopropanol and 15 mg/L Congo red solution. Samples were withdrawn every 30 min and analysed by UV Vis spectrometry. Ethylenediaminetetraacetic acid was used to scavenge for holes [98]. Isopropanol and Potassium dichromate were used to scavenge for hydroxyl and superoxide radicals, respectively [99]. As shown in Fig. 15, the addition of potassium dichromate resulted in a decrease in reaction efficiency by 76.01% confirming the superoxide is the main active species towards oxidative degradation of Congo red. Adam et al. [100] also made similar observations during their studies on degradation of Congo red. The addition of ethyleneaminetetraacetatic acid resulted in a decrease in extraction efficiency by 53% thereby indicating that $h^{+}$ holes also played a major role in the dye decolouration reaction. Isopropanol resulted in a 49.9% decrease in efficiency indicating that the hydroxyl radicals had a minor role whereas light only resulted in a decrease by 53.0% indicating that photolysis also played a role in the reaction.

**Figure 16:**
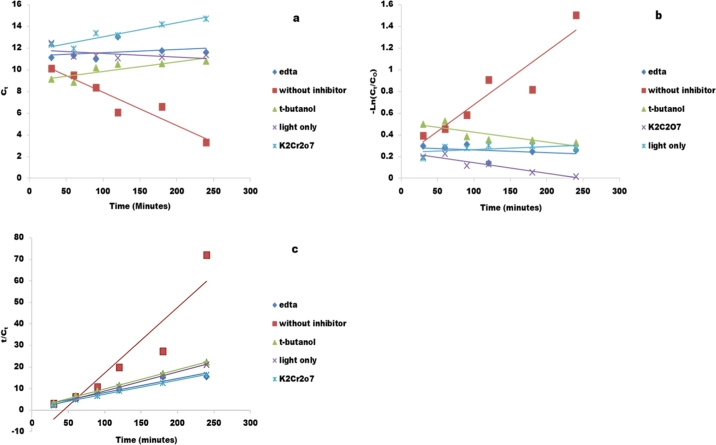
Kinetic modelling of the 15 mg/L Congo red inhibition reactions a) Pseudo zero order, b) Pseudo first order, c) Pseudo second order kinetic modelling.

The kinetics of the inhibition reaction were studied using reactions Pseudo zero order, Pseudo first order and Pseudo second order kinetic modelling and the results are shown in Fig. 16 a, b and c respectively and Table 6. Inhibition reactions for 1 mM Ethyleneaminetetraacetic acid, 1 mM t-butanol, light only without catalyst, and 1 mM potassium dichromate followed second order kinetics with rate constants of 0.0651, 0.0889, 0.0889 and 0.0652 min^−1^, respectively. This implies that both Congo red and the inhibitor played a role on the degradation reaction though the role's significance varied with inhibitor. Pseudo second order kinetics implies that the rate is proportional to the product of the concentration of the two reactants or the square of the molar concentration if the reactant is only one. The reaction which was carried out without inhibitor followed zero order kinetics with rate constant 0.0306 min^−1^. This means that the concentration of Congo red alone had no significant effect on the photodegradation reaction since some other species also played a role.

**Table 6:** Kinetic parameters for the inhibition reactions.

	Pseudo zero order		Pseudo first order		Pseudo second order	
Inhibitor	$K_{\text{app}}$	R^2^	$K_{\text{app}}$	R^2^	$K_{\text{app}}$	R^2^
	(min^−1^)		(min^−1^)		(min^−1^)	
*EDTA*	0.0029	0.089	0.003	0.0995	0.0651	0.9427
Without inhibitor	0.0306	0.9021	0.0049	0.873	0.3046	0.8681
t-butanol	0.0089	0.7284	0.009	0.7123	0.0889	0.9983
Light only	0.0033	0.244	0.003	0.2398	0.0889	0.9993
Potassium dichromate	0.0128	0.896	0.001	0.8819	0.0652	0.9967

When irradiated with visible light $P-ZrO_{2}CeO_{2}ZnO$ nanoparticles absorb the incident photons which are higher than the bandgap of the material. The electrons get excited in the valence band (VB) and go up into the conduction band (CB). At the same time an equal number of holes ($h^{+}$) are generated in the VB, electrons and holes are transferred between $ZrO_{2}$, $CeO_{2}$ and *ZnO*. Electrons jump from $ZrO_{2}$ to $CeO_{2}$ and *ZnO* in the conduction band. The holes are transferred from $ZrO_{2}$ to $CeO_{2}$ and *ZnO* in the valence band. The holes and electrons can take part in the chemical reaction which results in separation of photo induced electron hole pairs and a decrease of recombination rate in the catalyst [101]. A positive hole is formed when electrons reside in the conduction band as shown in Equation (18)

(18) $$P-ZrO_{2}CeO_{2}ZnO+hv\;⟶\;e_{CB}^{-}+h_{VB}^{+}$$

The excited electrons can also alternatively transfer from a conduction band of *ZnO* which has a lower fermi level and better electrical conductivity, Equations (19)–(21).

(19) $$ZnO+hv\;⟶\;e_{CB}^{-}+h_{VB}^{+}$$

(20) $$e_{CB}^{-}+ZrO_{2}\;⟶\;e^{-}$$

(21) $$e_{CB}^{-}+CeO_{2}\;⟶\;e^{-}$$

The generated photoelectrons will be scavenged by dissolved oxygen in water producing oxygen radicals, Equation (22).

(22) $$e^{-}+O_{2}\;⟶\;\cdot \;O^{2-}$$

The generated oxygen radicals combine with hydrogen ions to form $HOO^{\cdot }$, Equation (23).

(23) $$\cdot \;O^{2-}+H^{+}⟶\;HOO^{\cdot }$$

$HOO^{\cdot }$ combines with trapped electrons to generate hydrogen peroxide, Equation (24).

(24) $$HOO^{\cdot }+e^{-}+H^{+}\;⟶\;H_{2}O_{2}$$

Hydrogen peroxide also combines with trapped electrons to form hydroxyl radicals, Equation (25).

(25) $$H_{2}O_{2}+e^{-}\;⟶\;\cdot \;OH+OH^{-}$$

The positive holes on the valence band also react with water or the surface hydroxyl groups to form hydroxyl radicals. Equations (26)–(27).

(26) $$H_{2}O+H^{+}\;⟶\;\cdot \;OH+H^{+}$$

(27) $$OH^{-}+h^{+}\;⟶\;\cdot \;OH$$

The hydroxyl and superoxide radicals then react with Congo red to form carbon dioxide, water and other non-toxic products, Equation (28).

(28) $$\text{Congo red}+\cdot \;OH\text{ or }O_{2}^{.-}\;⟶\;CO_{2}+H_{2}O+\text{other non-toxic products}$$

**Figure 17:**
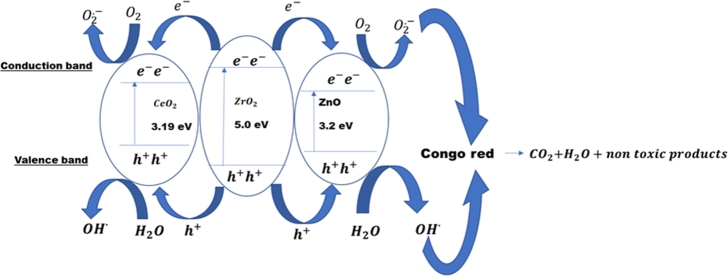
Proposed photocatalytic mechanism scheme of *P* − *ZrO*_2_*CeO*_2_*ZnO*.

The proposed reaction mechanism summarising the above discussed equations is shown in Fig. 17. Electrons are transferred within the metals in the photocatalyst and positive holes are created. The electrons are used to form oxygen and hydroxyl radicals which oxidizes Congo red to non-toxic compounds.

The effectiveness of the $P-ZrO_{2}CeO_{2}ZnO$ nanoparticles were compared with other catalyst using the degradation efficiency and rate constant as shown in Table 7. The photocatalyst compares well with other catalysts from previous studies.

**Table 7:** Photodegradation efficiency of catalysts over Congo red.

Photocatalyst	Light source	Degradation	Rate constant	Reference
		efficiency (%)	(min^−1^)	
*ZnO*	100 W UV light	53.1	0.0062	[102]
*Au*/*ZnO*		77.2	0.0196	
*Ag*/*ZnO*		81.6	0.0226	
*Pd*/*ZnO*		98.2	0.0576	
*Graphene* − *TiO*_2_	Sunlight	90	0.0546	[103]
*Fe* − *CeO*_2_	100 W tungsten lamp	96		[87]
*P*/*Bi*_3.84_*W*_0.16_*O*_6.24_	300 W	100	0.09744	[104]
	xenon lamp			
*MgTiO*_2_	150 W Tungsten	98	0.00162	[105]
*MnFe*_2_*O*_4_/*TA*/*ZnO*	128 W Xenon lamp	84.2	9.60	[9]
*Zn*_0.94_*Ni*_0.06_*S*	128 W		0.0255	[106]
*Zn*_0.90_*Cu*_0.10_*S*	Xenon		0.0338	
*Cd*,*Ba* − *CuO*	300 W Xenon arc lamp	98	0.012	[107]
*P* − *ZrO*_2_*CeO*_2_*ZnO*	20 W LED light	85.85	0.0069	This study

## 4 Conclusions

A green method for the synthesis of $P-ZrO_{2}CeO_{2}ZnO$ nanoparticles was established. The formation of the nanoparticles was confirmed through SEM and TEM analysis. Synthesis of nanoparticles was influenced by variables such as pH, dosage and metal concentration. There was interaction between factors such as metal concentration and volume ratio, plant dosage to volume ratio, plant dosage to metal concentration, pH and volume ratio, pH and metal concentration, pH and dosage during the synthesis stage. Information obtained from these experiments enabled the selection of optimum conditions. The conditions of the optimum $P-ZrO_{2}CeO_{2}ZnO$ nanoparticles sample obtained using the Taguchi design were pH 9, dosage 4 g/100 mL, metal concentration 0.05 M and volume ratio 1:4 The phytochemicals such as flavonoids, aldehydes, ketones, reducing sugars, were likely to have influenced the synthesis and stabilization of $P-ZrO_{2}CeO_{2}ZnO$ nanoparticles and were also identified by their functional groups using FT-IR analysis. The functional groups absorption peaks shifted or were totally absent in the nanoparticle band included those of the *OH*, C=O, and C−O. The$P-ZrO_{2}CeO_{2}ZnO$ nanoparticles showed high activity for Congo red degradation under domestic LED light irradiation in aqueous medium. Low catalyst concentration promoted an increase in the rate of reaction due to availability of active sites and high catalyst concentration resulted in lower rate of decolouration due to the accumulation of particles which led to dispersion and reduced penetration of light and subsequently limited decolourization. Low dye concentrations resulted in large removal efficiencies due to high absorption desorption equilibria. High dye concentrations result in more Congo red molecules, intermediates and photoproducts competing for absorption onto the active sites of the catalyst surface leading to an effective reduction in the reaction rate. The reaction requires a longer time to enable the catalyst to degrade optimum molecules and reach equilibrium. The reaction follows pseudo first order kinetics at lower concentration, pseudo second order kinetics at medium concentration and zero order kinetics at high concentrations of Congo red. The results of the photocatalytic studies reveal that superoxide, $h^{+}$ holes and light are the main determinants of the reaction mechanism for the efficient degradation of Congo red.

## Declarations

### Author contribution statement

Nichodimus Hokonya: Conceived and designed the experiments; Performed the experiments; Analyzed and interpreted the data; Contributed reagents, materials, analysis tools or data; Wrote the paper. Courtie Mahamadi: Conceived and designed the experiments; Analyzed and interpreted the data; Contributed reagents, materials, analysis tools or data; Wrote the paper. Netai Mukaratirwa-Muchanyereyi, Timothy Gutu, Caliphs Zvinowanda: Analyzed and interpreted the data; Contributed reagents, materials, analysis tools or data; Wrote the paper.

### Funding statement

This research did not receive any specific grant from funding agencies in the public, commercial, or not-for-profit sectors.

### Data availability statement

No data was used for the research described in the article.

### Declaration of interests statement

The authors declare no conflict of interest.

### Additional information

No additional information is available for this paper.
